# Generation of structurally novel short carotenoids and study of their biological activity

**DOI:** 10.1038/srep21987

**Published:** 2016-02-23

**Authors:** Se H. Kim, Moon S. Kim, Bun Y. Lee, Pyung C. Lee

**Affiliations:** 1 The Novo Nordisk Foundation Center for Biosustainability, Technical University of Denmark, Kogle Alle 6, 2970 Hørsholm, Denmark; 2 Department of Molecular Science and Technology and Department of Applied Chemistry and Biological Engineering, Ajou University, Woncheon-dong, Yeongtong-gu, Suwon 443-749, South Korea

## Abstract

Recent research interest in phytochemicals has consistently driven the efforts in the metabolic engineering field toward microbial production of various carotenoids. In spite of systematic studies, the possibility of using C_30_ carotenoids as biologically functional compounds has not been explored thus far. Here, we generated 13 novel structures of C_30_ carotenoids and one C_35_ carotenoid, including acyclic, monocyclic, and bicyclic structures, through directed evolution and combinatorial biosynthesis, in *Escherichia coli*. Measurement of radical scavenging activity of various C_30_ carotenoid structures revealed that acyclic C_30_ carotenoids showed higher radical scavenging activity than did DL-α-tocopherol. We could assume high potential biological activity of the novel structures of C_30_ carotenoids as well, based on the neuronal differentiation activity observed for the monocyclic C_30_ carotenoid 4,4′-diapotorulene on rat bone marrow mesenchymal stem cells. Our results demonstrate that a series of structurally novel carotenoids possessing biologically beneficial properties can be synthesized in *E. coli*.

Isoprenoids and their derivatives such as sterols, terpenes, dolichols, quinines, etc. are the most abundant class of secondary metabolites with diverse functions in microbial, plant, and animal metabolism^1^. The building block of isoprenoid derivative compounds is isopentenyl diphosphate (IPP), which is synthesized via two non-homologous pathways: the mevalonate pathway and the 1-deoxy-d-xylulose 5-phosphate/2-*C*-methyl-d-erythritol 4-phosphate (DOXP/MEP) pathway^2^,^3^. Terpenoids and carotenoids are the most industrially important isoprenoid derivatives that are used as cosmeceuticals, flavors, colorants, and pharmaceutical compounds.

Many efforts for microbial production of valuable compounds including isoprenoids and polyketides have been made using metabolic engineering^4^,^5^,^6^,^7^. Most of these studies have focused on providing an improved pool of precursors such as IPP in heterologous hosts including *E. coli* and yeasts. More recent studies have focused on the optimization of metabolic flux for enhanced target production to avoid accumulation of harmful intermediates and control rate-limiting steps in the heterologous host^8^. To this end, synthetic and systems biology approaches have been adapted to control pathway enzymes at both the transcriptional and translational levels with synthetic biological parts including engineered promoters, ribosome binding sites, and genetic circuits^9^,^10^.

Carotenoids are derivatives of isoprenoids that play diverse roles in nature^11^. Thus far, more than 700 carotenoids have been isolated and identified^12^. Carotenoids gained considerable research attention in the past owing to their usability as natural pigments; however, their high antioxidative and anticarcinogenic activities and potential use as cosmeceutical or pharmaceutical compounds have only been revealed more recently^13^,^14^,^15^,^16^. Animals obtain carotenoids through their diet, via consumption of fruits and vegetables. After oxidative cleavage of the dietary carotenoids (including xanthins), the bioactive cleavage products, such as apocarotenoids, are used in retinal formation and transcription system activation^17^. In addition, apocarotenoids are known to act as anticancer agents and cellular modulators of the retinoic acid receptors, retinoid X receptors, peroxisome proliferator-activated receptors, and estrogen receptors^17^. In a recent study, we proposed an anticancer mechanism of two major carotenoids, crocin and crocetin, found in the dried, dark red stigmas of *Crocus sativus*, which has been traditionally used since ancient times in Southwest Asia for the treatment of some diseases and as a flavoring and coloring agent^18^. These two carotenoids are C_20_ structures generated by the cleavage of C_40_ carotenoids, the most abundant carotenoid structures in nature including lycopene, β-carotene, and astaxanthin. Some bacteria produce C_50_ or C_30_ carotenoids; however, these are structurally less diverse than the C_40_ carotenoids. Until date, only few C_30_ carotenoid-producing natural sources are known, including *Staphylococcus aureus*, *Methylomonas* sp. 16a, and some strains of the genus *Bacillus*^19^,^20^,^21^,^22^.

Several studies successfully discovered novel carotenoids by applying a combinatorial biosynthesis approach with *in vitro* evolved carotenogenic enzymes^23^,^24^,^25^,^26^,^27^. *In vitro* evolution of key enzymes, which determine the structure of carotenoid in the early step, has led generation of novel series of carotenoids in *E. coli*^23^,^24^,^25^,^26^,^27^. Recently, Furubayashi and coworkers created non-natural C_50_ carotenoids through the assembly of moderately selective enzymes engineered by directed evolution in *E. coli*^28^. Enzymes commonly show strict substrate specificity; however, carotenogenic enzymes show relatively promiscuous substrate specificity giving rise to novel carotenoid structures in a heterologous host^29^. Phytoene synthase (CrtB), phytoene desaturase (CrtI), lycopene cyclase (CrtY), 4,4′-diapophytoene synthase (CrtM), and 4,4′-diapophytoene desaturase (CrtN) were evolved *in vitro*, allowing the biosynthesis of diverse carotenoids in *E. coli*^30^. Although extensive work has been performed on the diversification and identification of carotenoid structures, the existence of cyclic C_30_ carotenoids in nature has not been reported; 4,4′-diapotorulene was the first reported monocyclic C_30_ carotenoid structure biosynthesized by metabolically engineered *E. coli*^23^.

In this study, we created 13 structurally novel C_30_ carotenoids, including acyclic, monocyclic, and bicyclic structures, and one novel C_35_ carotenoid, through combinatorial biosynthesis with natural carotenoid biosynthetic enzymes from various microorganisms and the evolved enzymes, CrtN and CrtY, in *E. coli* (Fig. 1). Furthermore, we assayed the 2,2-diphenyl-1-picrylhydrazyl (DPPH)-scavenging and neuronal differentiative activities of the C_30_ carotenoids on rat bone marrow mesenchymal stem cells to explore their potential as bioactive compounds.

## Results

### Biosynthesis of structurally novel acyclic C_30_ carotenoids and a C_35_ carotenoid

Previously, CrtN mutant clones obtained by directed evolution showed altered desaturation activity in *E. coli*^30^. Similarly, we constructed a CrtN mutant library by error-prone PCR upon expression of the substrate, 4,4′-diapophytoene, in *E. coli*. We screened clones based on the color change to dark reddish, yellowish, and pale-yellowish colonies, depending on the number of conjugated double bonds (CDBs) in the chromophore of the carotene backbone, as compared to the reddish wild-type colonies. Their carotenoid profiles were monitored by HPLC to select CrtN_r_, CrtN_y_, and CrtN_z_ mutant clones showing increased pathway selectivity for 4,4′-diapolycopene, 4,4′-diaponeurosporene, and 4,4′-diapo-ζ-carotene, respectively (peaks 1, 2, and 3 in Fig. 2a). Unfortunately, we failed to determine the structure of the CrtN mutants due to the lack of CrtN structural data, even though we performed site-directed mutagenesis (Supplementary Fig. S1) and docking prediction analysis.

In an attempt to create novel carotenoid structures, we first extended the wild-type 4,4′-diapolycopene pathway (pACM-M_SA_-N_SA_) using two wild-type enzymes, C_40_ spheroidene monooxygenase (CrtA_RC_) and C_40_ 1-hydroxycarotenoid 3,4-desaturase (CrtD_RC_) from *Rhodobacter capsulatus*, resulting in the generation of two new peaks in the HPLC profile (peaks 4 and 5 in Fig. 2b). Based on combined analysis of retention time, UV/Vis absorption, and mass spectra, peaks 4 and 5 were assigned to the acyclic C_30_ carotenoids 4,4′-diaponeurosporen-6,6′-dione and 4,4′-diapoplycopene-6,6′-dione, respectively (Fig. 2b). Especially, the broadened UV/Vis absorption spectrum and longer λ_max_ (467 nm) observed for the compound of peak 5 supported the presence of 11 fully CDBs and two ketone groups in its structure^31^.

Next, the 4,4′-diapolycopene pathway was extended with C_50_ lycopene elongase (CrtEb), which was cloned from *Corynebacterium glutamicum*. CrtEb catalyzes the sequential addition of two IPPs into the natural substrate lycopene to produce C_45_ nonaflavuxanthin and C_50_ flavuxanthin. Extension of the 4,4′-diapolycopene pathway with CrtEb resulted in the production of an elongated acyclic C_35_ carotenoid, 4,4′-diapononaflavuxanthin (peak 6 in Fig. 2c), assigned on the basis of combined structural analysis as described above.

### Biosynthesis of structurally novel cyclic C_30_ carotenoids

**Figure 1:**
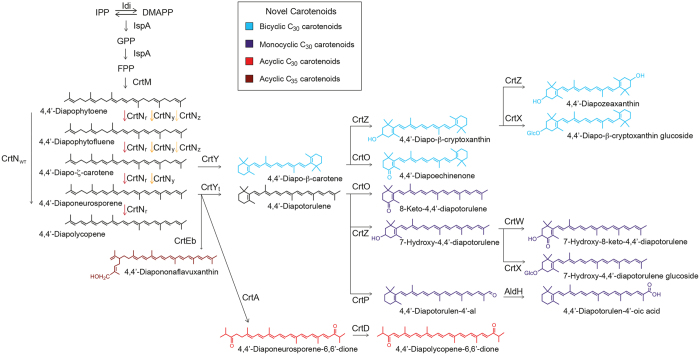
Carotenoid biosynthesis pathway diversified through *E. coli*. Each structure was classified.

Next, we aimed to create cyclic C_30_ carotenoids by introducing various bacterial C_40_ lycopene cyclases (CrtY) into the redesigned C_30_ carotenoid biosynthesis pathways. As β-ionone ring formation in C_30_ carotenoids requires the saturated C4-C5 and C4′-C5′ bonds of a carotene backbone, we chose the redesigned 4,4′-diapo-ζ-carotene (pACM-M_SA_-N_zSA_) and 4,4′-diaponeurosporene (pACM-M_SA_-N_ySA_) pathways (Fig. 1). First, expanding upon the redesigned 4,4′-diaponeurosporene pathway, we expressed each C_40_ CrtY from *Salinibacter ruber* (CrtY_SR_), *C. glutamicum* (CrtY_CG_), *Brevibacterium linens* (CrtY_BL_), *Pantoea ananatis* (CrtY_PN_), and *Pantoea agglomerans* (CrtY_PA_) to investigate their non-natural substrate promiscuity to form one β-ionone ring in acyclic C_30_ 4,4′-diaponeurosporene. Among them, CrtY_BL_ and CrtY_PA_ produced new HPLC peaks, while the others did not show noticeable differences when compared to control cells expressing empty vector (Fig. 3a). Based on combined structural analysis, we speculated that CrtY_BL_ catalyzed the cyclization of one saturated end of non-natural substrate 4,4′-diaponeurosporene, generating structurally novel monocyclic C_30_ 4,4′-diapotorulene (peak 3 in Fig. 3a). To enhance the selectivity toward the monocyclic 4,4′-diapotorulene pathway, CrtY_BL_ was evolved by error-prone PCR and some mutant CrtY_tBL_ enzymes showing higher selectivity toward 4,4′-diapotorulene (Supplementary Fig. 2) were selected for pathway extension (see next section). Interestingly, unlike the high selectivity of CrtY_BL_ for 4,4′-diapotorulene, CrtY_PA_ showed low specificity toward non-natural short substrates 4,4′-diaponeurosporene and 4,4′-diapo-ζ-carotene, and low catalytic efficiency. We hypothesized that CrtY_PA_ cyclized each saturated end of the non-natural substrate 4,4′-diapo-ζ-carotene and one saturated end of the non-natural substrate 4,4′-diaponeurosporene, creating bicyclic C_30_ 4,4′-diapo-β-carotene (peak 4 in Fig. 3a) and monocyclic C_30_ 4,4′-diapotorulene, respectively, albeit at very small amounts. Notably, the formation of bicyclic 4,4′-diapo-β-carotene indicated that CrtY_PA_ had an affinity for 4,4′-diapo-ζ-carotene, one of pathway intermediates in the redesigned 4,4′-diaponeurosporene pathway (Fig. 1).

Next, to selectively increase bicyclic 4,4′-diapo-β-carotene production, we coexpressed CrtY_BL_, CrtY_PA_, and mutant CrtY_tBL_ with the redesigned 4,4′-diapo-ζ-carotene pathway. As expected from the observed affinity of CrtY_PA_ for 4,4′-diapo-ζ-carotene, CrtY_PA_ produced 4,4′-diapo-β-carotene with high selectivity (Fig. 3b). Unexpectedly, CrtY_BL_ also produced a small amount of bicyclic 4,4′-diapo-β-carotene, which was not detected upon coexpression with 4,4′-diaponeurosporene, suggesting that the relative substrate concentration significantly influences the product profile of CrtY_BL_. Mutant CrtY_tBL_ produced 4,4′-diapo-β-carotene and 4,4′-diapotorulene at a similar ratio, indicating that mutant CrtY_tBL_ had a virtually equal substrate affinity for 4,4′-diapo-ζ-carotene and 4,4′-diaponeurosporene.

### Biosynthesis of structurally novel monocyclic C_30_ carotenoids harboring a modified β-ionone ring

Two β-ionone ring moiety-modifying enzymes, C_40_ carotene ketolase (CrtO_SY_) from *Synechocystis* sp. PCC 6830 and C_40_ β-carotene hydroxylase (CrtZ_PA_) from *P. agglomerans*, were individually coexpressed with the 4,4′-diapotorulene pathway (pACM-M_SA_-N_ySA_-Y_tBL_) to create structurally novel monocyclic C_30_ carotenoids. Combined structural analysis revealed that CrtZ_PA_ catalyzed the hydroxylation of the β-ionone ring in the non-natural substrate 4,4′-diapotorulene, creating 7-hydroxy-4,4′-diapotorulene (peak 4 in Fig. 4a), while CrtO_SY_ catalyzed the addition of a ketone group into the β-ionone ring, creating 8-keto-4,4′-diapotorulene (peak 3 in Fig. 4a).

The redesigned 7-hydroxy-4,4′-diapotorulene pathway (pACM-M_SA_-N_ySA_-Y_tBL_-Z_PA_) was further extended by individually coexpressing C_40_ carotene ketolase (CrtW_NO_) from *Nostoc* sp. PCC 7120 and C_40_ zeaxanthin glucosyltransferase (CrtX_PA_) from *P. agglomerans*. The results indicated that CrtX_PA_ catalyzed the glycosylation of the hydroxyl-β-ionone ring in the non-natural substrate 7-hydroxy-4,4′-diapotorulene, creating 7-hydroxy-4,4′-diapotorulene glucoside (peak 6 in Fig. 4a), while CrtW_NO_ catalyzed the addition of a ketone group into the hydroxyl-β-ionone ring, creating 7-hydroxy-8-keto-4,4′-diapotorulene (peak 5 in Fig. 4a).

### Biosynthesis of structurally novel monocyclic C_30_ carotenoids with a modified acyclic end

Next, we aimed to create novel monocyclic C_30_ carotenoid structures by modifying the acyclic end of the carotene backbone. We first coexpressed C_30_ 4,4′-diaponeurosporene oxidase (CrtP_SA_) from *S. aureus*^32^ with the redesigned 4,4′-diapotorulene pathway. As expected from the reported low specificity of CrtP_SA_^32^, novel C_30_ 4,4′-diapotorulen-4′-al (peak 8 in Fig. 4b) was produced, and 4,4′-diaponeurosporen-4′-al accumulated (peak 7 in Fig. 4b). Notably, CrtP_SA_ could compete with Y_tBL_ for 4,4′-diaponeurosporene substrate for the production of 4,4′-diaponeurosporen-4′-al and 4,4′-diapotorulene, respectively, which were further modified by the other competing enzymes. The reaction sequence for 4,4′-diapotorulen-4′-al production is unclear, but based on the fact that 4,4′-diapotorulene was not detectable on HPLC, we postulate that Y_tBL_ would first produce 4,4′-diapotorulene, to which an aldehyde group was added by CrtP_SA_, resulting in the formation of 4,4′-diapotorulen-4′-al.

For further extension of the redesigned 4,4′-diapotorulen-4′-al pathway (pACM-M_SA_-N_ySA_-P_SA+_pUCM-Y_tBL_), C_30_ 4,4′-diaponeurosporene aldehyde dehydrogenase (AldH_SA_) from *S. aureus*^32^ was expressed. The results suggested that AldH_SA_ catalyzed the oxidation of non-natural substrate 4,4′-diapotorulen-4′-al, creating novel C_30_ 4,4′-diapotorulen-4′-oic acid (peak 10 in Fig. 4b). These novel monocyclic C_30_ structures could be used as substrates for carotenoid cleavage dioxygenases for the production of novel apocarotenoids^33^. ^1^H NMR analysis of 4,4′-diapotorulene was performed to provide structural evidence of 4,4′-diapotorulene derivatives produced in *E. coli* (Supplementary Fig. 3).

### Biosynthesis of structurally novel bicyclic C_30_ carotenoids

Next, we attempted to extend the bicyclic C_30_ 4,4′-diapo-β-carotene pathway. Similar to the extension of the monocyclic 4,4′-diapotorulene pathway, we individually coexpressed C_40_ CrtO_SY_ and CrtZ_PA_ with the redesigned 4,4′-diapo-β-carotene pathway (pACM-M_SA_-N_zSA_-Y_PA_). Combined structural analysis indicated that CrtZ_PA_ catalyzed the hydroxylation of the β-ionone ring at each end of the non-natural substrate 4,4′-diapo-β-carotene, creating C_30_ 4,4′-diapo-β-cryptoxanthin (peak 5 in Fig. 5a) through one-step hydroxylation, and a very tiny amount of C_30_ 4,4′-diapozeaxanthin (peak 6 in Fig. 5a) through two-step hydroxylation. Unlike the two-step reactions of CrtZ_PA_ on the two β-ionone rings, CrtO_SY_ only catalyzed one addition of a ketone group into one β-ionone ring in the non-natural substrate 4,4′-diapo-β-carotene, creating C_30_ 4,4′-diapoechinenone (peak 7 in Fig 5a). The detection of a tiny amount of 4,4′-diapozeaxanthin and the absence of the expected structure 4,4′-diapocanthaxanthin suggested that the catalytic activity of C_40_ carotenoid-modifying enzymes towards the non-natural short substrate 4,4′-diapo-β-carotene was lower than that towards the other non-natural short substrate 4,4′-diapotorulene.

Finally, we coexpressed C_40_ CrtX_PA_ with the redesigned 4,4′-diapo-β-cryptoxanthin pathway (pACM-M_SA_-N_zSA_-Y_tBL_-Z_PA_). On the basis of combined structural analysis, we hypothesized that CrtX_PA_ catalyzed the glycosylation of a hydroxyl-β-ionone ring in the non-natural substrate 4,4′-diapo-β-cryptoxanthin, creating C_30_ 4,4′-diapo-β-cryptoxanthin glucoside (peak 8 in Fig. 5b).

Taken together, we created 14 structurally novel carotenoids in recombinant *E. coli*; their mass spectra are shown in Supplementary Fig. 4.

### Radical scavenging activity of structurally novel C_30_ carotenoids

Most carotenoids typically have antioxidative activity^13^. Recently, carotenoids are gaining considerable research attention, along with the increasing interest in the role of dietary phytochemicals in human health^34^. While antioxidative activities of natural C_40_ carotenoids have been well analyzed, C_30_ carotenoids have been hardly studied because they are rare in nature and are absent in plants. Therefore, we investigated the radical scavenging activity of five purified, novel C_30_ carotenoids, including acyclic, monocyclic, and bicyclic structures, as well as two previously reported C_30_ carotenoids (4,4′-diapolycopene and 4,4′-diaponeurosporene) using the DPPH free radical method^35^,^36^. DL-α-tocopherol was included as a control compound^37^. Scavenging reactions were found to be optimal when molar ratios of carotenoid:DPPH between 1:10 and 1:20 were used (data not shown). As expected from the reported antioxidant activities of various C_40_ carotenoid structures, C_30_ carotenoids containing higher numbers of CDBs tended to exhibit higher radical scavenging activity than those with fewer CDBs (Fig. 6a), e.g., 4,4′-diapolycopene (13 CDBs) vs. 4,4′-diaponeurosporene (11 CDBs). Notably, 4,4′-diapolycopene-4,4′-dial (13 CDBs+2 aldehyde groups) was the most efficient scavenger, followed by 4,4′-diapolycopene (13 CDBs), 4,4′-diaponeurosporenoic acid (11 CDBs+1 carboxylic group), 4,4′-diaponeurosporenoic acid (11 CDBs+1 aldehyde group), and 4,4′-diaponeurosporene (11 CDBs) (Table 1). Cyclic C_30_ carotenoids, 4,4′-diapotorulene and 4,4′-diapo-β-carotene, showed lower antioxidative activity than acyclic C_30_ carotenoids, and even lower than the antioxidant control DL-α-tocopherol (Fig. 6a, Table 1). Collectively, the number of CDBs seemed to be one of the most important factors influencing the antioxidative activity of C_30_ carotenoids, and C_30_ carotenoids having a carboxyl group at the end of the backbone had slightly higher antioxidative activities than those with the same number of CDBs but with an aldehyde end group.

### Neuronal differentiation activity of structurally novel C_30_ carotenoid 4,4′-diapotorulene

Retinoic acid, an important signaling molecule, has been extensively investigated for its role in inducing neuronal differentiation from various progenitor cells^38^,^39^. Because 4,4′-diapotorulen-4′-oic acid is structurally similar to retinoic acid, we assumed that it would also have similar biological activity. However, since the amount of purified 4,4′-diapotorulen-4′-oic acid produced by recombinant *E. coli* was not sufficient for neuronal differentiation and cytotoxicity assays, 4,4′-diapotorulene was selected for the neuronal differentiation assay in rat bone marrow mesenchymal stem cells (rBMSCs)^40^.

Cytotoxicity of 4,4′-diapotorulene in the rBMSCs was dose-dependent, and approximately 20% of the cells were died after 7 days of treatment with the highest concentration (50 μM) of 4,4′-diapotorulene as compared to control cells (Supplementary Fig. 5). When rBMSCs were treated with varying concentrations of 4,4′-diapotorulene (2, 10, 20, and 50 μM), rBMSCs exhibited bipolar/multipolar or web-shaped morphology characteristic of neuronal cells after a 24-h treatment (Fig. 6b). With longer incubation time (4 and 7 days), the morphological changes enhanced in a dose-dependent manner. These findings indicated the potential neuronal differentiation activity of monocyclic C_30_ 4,4′-diapotorulene on rBMSCs.

## Discussion

Metabolic engineering enables the creation of non-natural pathways for novel structures of biotechnological importance, especially by exploiting the promiscuity of biosynthetic pathway enzymes and directed evolution^41^,^42^,^43^,^44^. Recent research interest in phytochemicals has consistently driven the efforts in the metabolic engineering field toward microbial production of various isoprenoids. In spite of systematic studies on the microbial production of carotenoids, similar to other isoprenoids such as terpenoids, the possibility of using C_30_ carotenoids, which have not been reported in plant sources, as biologically functional compounds has not been explored thus far. Our study sheds light on the generation of a new series of short C_30_ carotenoid structures as well as on their potential biological properties such as radical scavenging activity and neuronal differentiation activity. Carotenoids including apo-carotenoids are well known as antioxidative compounds, also interested by their other beneficial biological activities including anticancer activity^17^. Even though some studies have been made to understand relationship between structure of carotenoids and their anticancer effect, still there is a lack of knowledge on how carotenoids are transferred and modified into apo-carotenoids in the cell^45^,^46^. Nevertheless, it is known that complex structures of carotenoids with longer CDBs and/or more functional groups show higher antioxidant activity, which was consistent with our radical scavenging assay (Fig. 6a). Microbial production of apo-carotenoids of biotechnological interest using carotenoid cleavage enzymes is not promising due to low yield and productivity^33^. Thus, our strategy to produce short C_30_ carotene-aldehyde/acid derivatives can be served as an alternative method without employing carotenoid cleavage enzymes. Furthermore, as 4,4′-diapotorulene showed neuronal differentiation activity (Fig. 6b) like retinoic acid, an important apo-carotenoid in cell development^38^, 4,4′-diapotorulene derivatives could open new opportunities for developing bioactive compounds.

**Figure 2:**
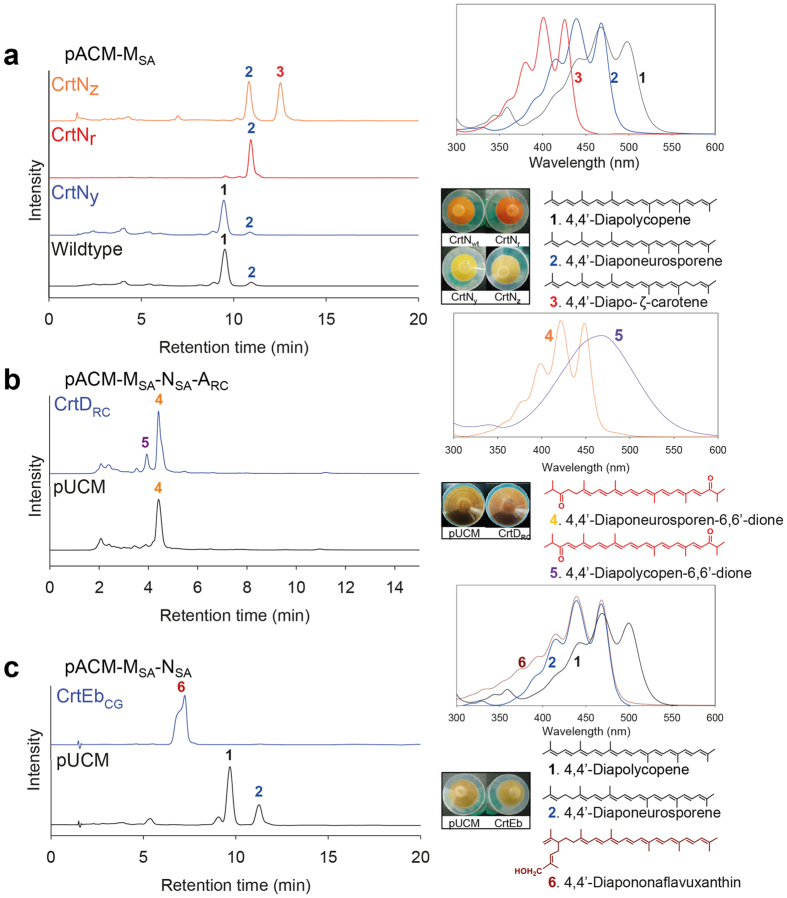
Analysis of C_30_and C_35_ acyclic carotenoids produced by engineered *E. coli* cells. (**a**) HPLC profiles of the C_30_ acyclic carotenoids pathway reconstructed in *E. coli* expressing background pACM-M_SA_ and wild-type and mutant CrtN. (**b**) HPLC profiles of the C_30_ carotenoids pathway reconstructed in *E. coli* expressing pACM-M_SA_-N_SA_-A_RS_ and CrtD_RC_. (**c**) HPLC profiles of the C_35_ acyclic carotenoid pathway reconstructed in *E. coli* expressing pACM-M_SA_-N_SA_ and CrtEb_GC_. UV/Vis absorption spectra for compounds corresponding to individual peaks are shown in the upper-right panels. Cell pellets and identified carotenoid structures are shown in the lower-right panel.

**Figure 3:**
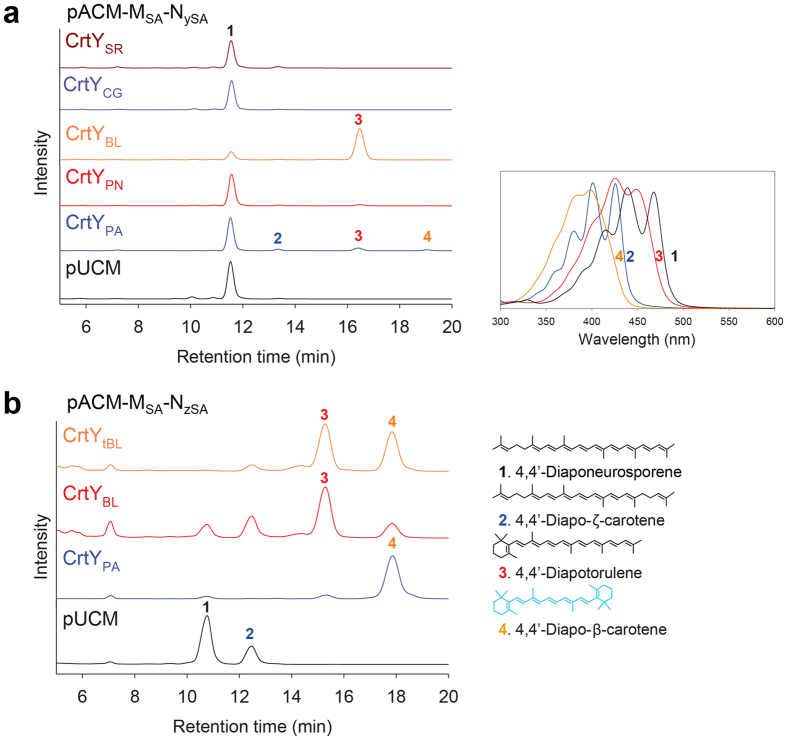
Functional comparison of CrtYs from various sources in *E. coli* expressing the 4,4′-diaponeurosporene or 4,4′-diapo-ζ-carotene pathway. (**a**) Each C_40_ CrtY from a different source was coexpressed with the 4,4′-diaponeurosporene pathway, pACM-M_SA_-N_ySA_, to compare its activity on the C_30_ backbone. (**b**) *In vivo* activity of mutant CrtY_tBL_, wild-type CrtY_BL_, and CrtY_PA_ on 4,4′-diapo-ζ-carotene was compared in *E. coli* expressing pACM-M_SA_-N_zSA_. UV/Vis absorption spectra for compounds corresponding to individual peaks are shown in the upper-right panel. Identified carotenoid structures are shown in the lower-right panel.

**Figure 4:**
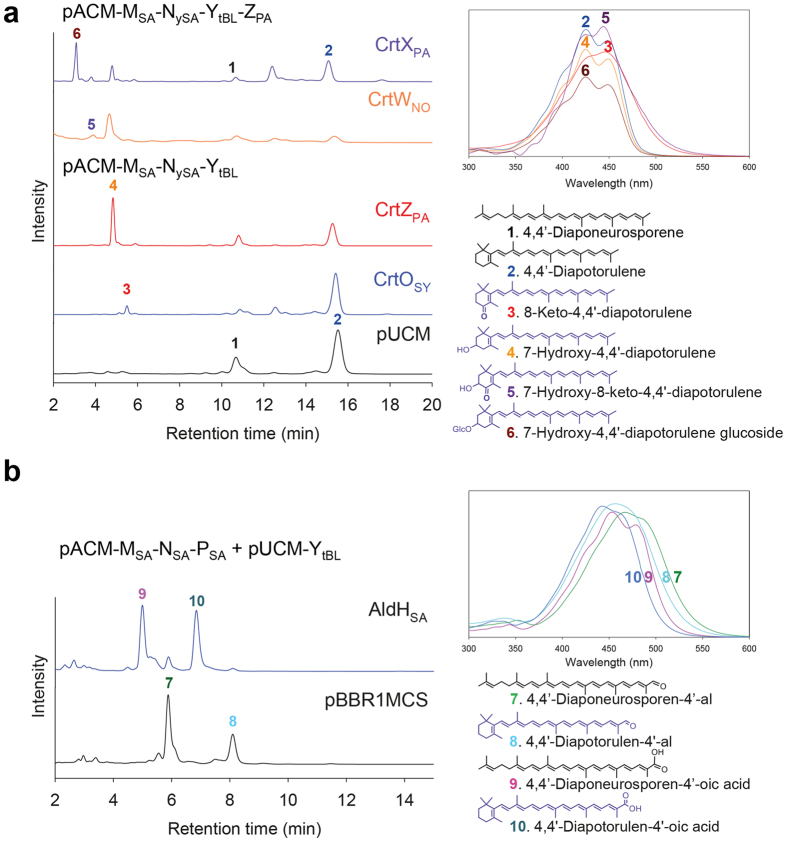
Analysis of C_30_ monocyclic carotenoids produced by engineered *E. coli* cells. (**a**) HPLC profiles of the C_30_ monocyclic carotenoids pathway reconstructed in *E. coli* expressing background pACM-M_SA_-N_ySA_-Y_tBL_ or pACM-M_SA_-N_ySA_-Y_tBL_-Z_PA_ and C_40_ carotenoid modifying enzymes (CrtX_PA,_ CrtW_NO,_ CrtZ_PA_ and CrtO_SY_). (**b**) HPLC profiles of the C_30_ monocyclic carotenoids pathways reconstructed in *E. coli* expressing pACM-M_SA_-N_SA_-P_SA_ with CrtY_tBL_ and AldH_SA_. UV/Vis absorption spectra for compounds corresponding to individual peaks are shown in the upper-right panels. Identified carotenoid structures are shown in the lower-right panels.

**Figure 5:**
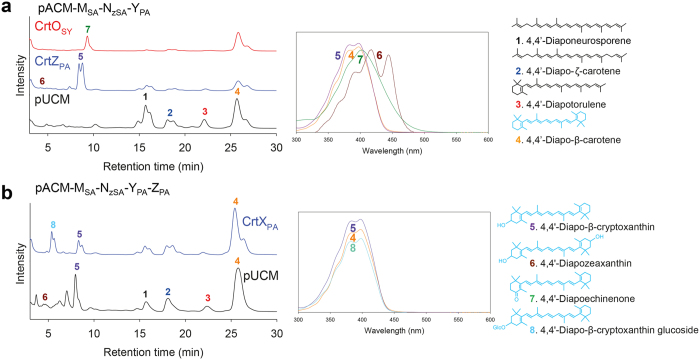
Analysis of C_30_ bicyclic carotenoids produced by engineered *E. coli* cells. (**a**) HPLC profiles of the C_30_ bicyclic carotenoids pathway reconstructed in *E. coli* expressing background pACM-M_SA_-N_zSA_-Y_PA_ and C_40_ carotenoid modifying enzymes (CrtO_SY_ and CrtZ_PA_). Insets: absorption spectra for individual peaks. (**b**) HPLC profiles of the C_30_ bicyclic carotenoids pathway reconstructed in *E. coli* expressing pACM-M_SA_-N_zSA_-Y_PA_-Z_PA_ and CrtX_PA_. UV/Vis absorption spectra for compounds corresponding to individual peaks are shown in the middle panels. Identified carotenoid structures are shown in the right panels.

**Figure 6:**
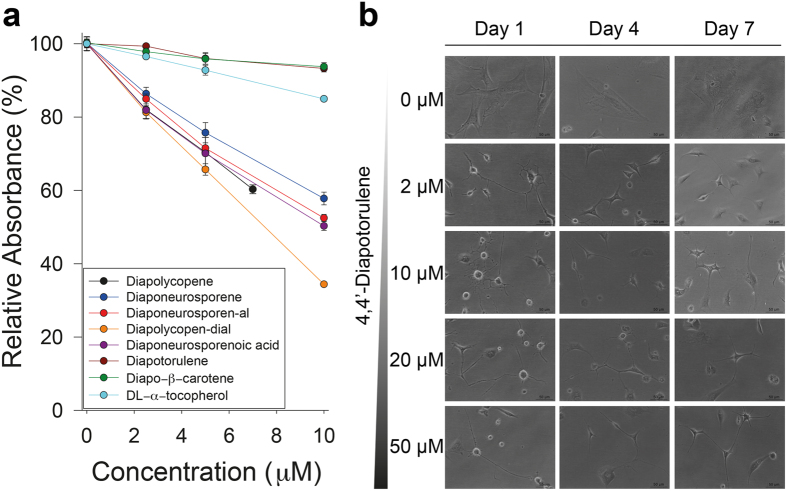
Comparison of DPPH scavenging activity of various C_30_ carotenoids and morphological changes of rBMSCs treated with varying concentrations of 4,4′-diapotorulene for 7 days. (**a**) DL-α-tocopherol was used as a control antioxidant. Values are represented as the mean ± SD from three independent experiments. (**b**) Cells were treated with varying concentrations of 4,4′-diapotorulene and observed under the microscope for 7 days. Scale bar represents 50 μm.

Directed evolution of enzymes in early steps in the biosynthesis pathway and combinatorial biosynthesis is a powerful tool for generating diverse novel structures in microbes as described in Introduction section. In this study, CrtN was evolved to optimize 4,4′-diaponeurosporene or 4,4′-diapo-ζ-carotene biosynthesis pathway (Fig. 2a), which could be cyclized into non-natural cyclic C_30_ carotenoids. Directed evolution of CrtY from *B. linens* successfully drove cyclization reaction of 4,4′-diaponeurosporene into 4,4′-diapotorulene (Fig. 3a). Through combinatorial biosynthesis by using different CrtY enzymes^47^, we achieved selective production of 4,4′-diapo-β-carotene from 4,4′-diapo-ζ-carotene (Fig. 3b). After we selectively optimized C_30_ carotenoid biosynthetic pathways using mutant CrtN and CrtY enzymes, combinatorial biosynthesis with diverse C_40_ and C_50_ carotenoid-modifying enzymes found in nature (Supplementary Fig. 6) yielded one acyclic C_35_ carotenoid and two acyclic, six monocyclic, and five bicyclic C_30_ carotenoids in recombinant *E. coli* (Fig. 1). Because new types of carotenoid enzymes including carotenoid cleavage enzymes, which can produce biologically more functional apo-carotenoids are continually being discovered and characterized, more structurally novel C_30_ carotenoids could be generated by using our strategies in the near future, as we had shown in the previous report^33^. The expanding pool of structurally novel short carotenoids will open up the possibilities for discovery of bioactive compounds, which are potential candidates for drug development and cosmetic applications. However, since this is the first report on the generation of novel C_30_ and C_35_ carotenoid structures, more detailed and comprehensive investigation of the biological and physiological activity of C_30_ carotenoids is warranted. To address this, more quantitative and systems-level studies are needed to selectively direct the metabolic flux towards the formation of novel structures and increase titer of these novel structures in *E. coli*. Finally, we believe that this work can serve as a basis for investigators handling various carotenoids as candidates of biological active compounds in cosmetics, pharmaceutical, feeds, and fine chemical industries.

## Materials and Methods

### Bacterial strains, plasmids, and growth conditions

The bacterial strains and plasmids used in this study are listed in Supplementary Table 1. For gene cloning, the *E. coli* XL1-Blue strain was grown at 37 °C in Luria-Bertani (LB) medium on a rotary shaker at 250 rpm. For carotenoid production, the *E. coli* SURE strain^29^ was grown at 30 °C in Terrific Broth (TB) medium on a rotary shaker at 250 rpm, except for pBBR-aldH_SA_, which was expressed in XL1-Blue because of kanamycin resistance of the SURE strain. Chloramphenicol (50 μg/mL), ampicillin (100 μg/mL), and/or kanamycin (30 μg/mL) (Sigma) were added as required. *P. agglomerans* KCTC 2479, *R. capsulatus* KCTC 2583, and *C. glutamicum* KCTC 1445 were cultivated in LB medium at 30 °C on a rotary shaker at 250 rpm. *S. ruber* DSMZ 13855 was grown in medium consisting of 195 g of NaCl, 34.6 g of MgCl_2_, 49.5 g of MgSO_4_, 1.25 g of CaCl_2_, 5 g of KCl, 0.25 g of NaHCO_3_, 0.625 g of NaBr, and 0.1 g of yeast extract per 1 L of double-distilled water at 37 °C for 7 days.

### Gene cloning and construction of C_30_ carotenogenic gene modules

The genes *crtY* (encoding lycopene cyclase), *crtX* (glucosyltransferase), and *crtD* (carotene 3,4-desaturase) were PCR-amplified from genomic DNA of *S. ruber*, *P. agglomerans* KCTC 2479, and *R. capsulatus* KCTC 2583, respectively, using gene-specific primers (Supplementary Table 2). The *crtEb* (lycopene elongase) and *crtY* (carotene cyclase) genes were amplified from *C. glutamicum* KCTC 1445 genomic DNA using gene-specific primers. The PCR products were cloned into pUCM vector^29^, resulting in pUCM-X_AB_ (where pUCM indicates the vector used, X represents the gene name, and the subscript _AB_ in X represents the gene source microorganism; see Supplementary Table 1). C_30_ carotenogenic gene modules were constructed as described previously^32^. Briefly, a gene was subcloned from pUCM-X_AB_ into pACYC184 by amplifying the gene together with a modified constitutive *lac*-promoter, resulting in pACM-X_AB_ (note: sequential assembly of a gene Y_AB_ into a module results in pACM-X_AB_-Y_AB_, for example). PCR amplification was carried out using a DNA Engine Thermal Cycler (Bio-Rad; Hercules, CA, USA) with Vent polymerase (New England Biolabs; Beverly, MA, USA) for cloning, with *Taq* polymerase (Intron Biotechnology; Seoul, Korea) for random mutagenesis, and, with pfuUltra II fusion polymerase (Agilent Technologies; Palo Alto, CA, USA) for site-directed mutagenesis. Restriction enzymes and T4 DNA ligase were all purchased from New England Biolabs.

### Directed evolution of CrtN and CrtY

A mutant library was constructed by error-prone PCR with *Taq* polymerase by increasing the MgCl_2_ concentration (up to 2.5 mM) and altering the dNTP ratio (ATP:TTP:CTP:GTP, 1:1:1:4). Error-prone PCR of *crtN* or *crtY* in pUCM was carried out under error-prone conditions with plasmid-specific PCR primers (5′-CCGACTGGAAAGCG-3′ and 5′-CGGTGTGAAATACCG-3′) flanking the gene and promoter. The PCR products of *crtN* and *crtY* were cloned into the pUCM vector and electrotransformed into recombinant *E. coli* XL1-Blue harboring pACM-M_SA_ and pACM-M_SA_-N_SA_, respectively. Colonies were screened visually on agar plates for color variants after 24 h of incubation (or until color developed) at room temperature. Selected colonies were aerobically grown in TB (4 mL) overnight, pelleted, and repeatedly extracted with 2–4 mL acetone. Thin-layer chromatography (TLC) was carried out using a 100% hexane solvent system for initial screening of positive mutant clones. For discrimination of false positive clones, plasmids having mutant a gene were isolated from positive clones and then retransformed into fresh competent *E. coli* cells harboring pACM-M_SA_ or pACM-M_SA_-N_SA_. Sequences of selected *crtN* and *crtY* clones were confirmed by Sanger sequencing (Macrogen; Seoul, Korea).

### Isolation of carotenoids

Cells were pelleted by centrifugation (4 °C, 4000 rpm), and carotenoids were repeatedly extracted from the pellets with 15 mL acetone until all visible pigments were removed from the pellets. The colored supernatants were pooled after centrifugation (4 °C, 4000 rpm) and concentrated to 5–10 mL using an EZ-2 *Plus* centrifugal evaporator (Genevac Inc.; Vally Center, NY, USA). An equal volume of ethyl acetate (EtOAc) was added to the concentrated solution that was re-extracted after adding an equal volume of 5 N NaCl solution for salting out. The upper phase, containing the carotenoids, was collected, washed with distilled water, passed through a sodium sulfate column (anhydrous, Bio Basic Inc; ON, Canada), and completely dried using the EZ-2 *Plus* evaporator. The dried samples were stored at −80 °C until further use.

For purification, the isolated carotenoids were dissolved in 0.2 mL of EtOAc, filtered (0.45-μm GHP membrane, Pall Corporation; NY, USA), and loaded onto a silica gel column, which was pre-equilibrated with a 100% hexane or a 9:1 hexane/EtOAc solvent system. Carotenoids were eluted using the same solvent system with an increasing gradient of EtOAc. Each of the eluted carotenoids was concentrated into a small volume, loaded onto a TLC silica gel plate (Merck Millipore, Billerica, MA, USA), and developed with solvent systems comprising hexane/EtOAc (9:1, v/v) or hexane/EtOAc/methanol (9:1:3, v/v/v). Each carotenoid band was scraped from the TLC plate, followed by elution with methanol. Glassware was used for each step, and stray light was blocked with aluminum foil. Purified C_30_ carotenoids were quantified using a spectrophotometer (SpectraMax Plus^384^, Molecular Devices; CA, USA) by comparison with the extinction coefficient of known, similar structures of C_40_ carotenoids^48^.

### Structural analysis of carotenoids

An aliquot (10–20 μL) of the collected fraction or crude extracts was applied to a ZORBAX Eclipse XDB-C18 column (4.6 mm × 150 mm or 4.6 mm × 250 mm, 5.0 μm; Agilent Technologies; Santa Clara, USA) and eluted under isocratic conditions with a solvent system (acetonitrile:methanol:isopropanol, 80:15:5) at a flow rate of 1 mL/min using an Agilent 1200 HPLC system equipped with a photodiode array detector (Agilent Technologies). Chromatograms were recorded at wavelengths of 470, 440, and 400 nm to trace the elution of linear, monocyclic, and bicyclic C_30_ carotenoids, respectively. The mass fragmentation spectra were monitored using both negative and positive ion modes in the mass range of *m/z* 200 to 600 on a liquid chromatography–mass spectrometer (LC/MS; Agilent 6150) equipped with an atmospheric pressure chemical ionization ion source (Agilent Technologies). The HPLC retention times, UV/Vis absorption spectra, and mass fragmentation spectra were all combined for the structural identification of the C_30_ carotenoids. For NMR analysis of 4,4′-diapotorulene, purified 4,4′-diapotorulene was dissolved in CD_3_OD and the ^1^H NMR (600 MHz) spectra were recorded on a Bruker Avance 600 system.

### DPPH radical scavenging assay

The 1,1-diphenyl-2-picrylhydrazyl (DPPH) radical scavenging assay for purified C_30_ carotenoids was performed as described previously^37^ with a minor modification. Buffered methanol was prepared by mixing 40 mL of 0.1 M acetate solution (pH 5.5) with 60 mL of methanol. Assays were initiated by adding 250 μL of 0.2 M DPPH solution into 200 μL of buffered methanol containing 50 μL of 100 μM carotenoid, and incubated in the dark for 1 h. The absorbance at 517 nm was measured using a UV/Vis spectrophotometer (SpectraMax Plus^384^) against a blank consisting of the reaction mixture without DPPH. dl-α-tocopherol was included as a control. Experiments were performed in triplicate and the data are presented as mean ± standard deviation (SD). IC_50_ values were calculated after curves were fitted using SigmaPlot 11.0.

### Cytotoxicity tests and neurogenesis of rBMSCs

4,4′-Diapotorulene was dissolved in DMSO at a concentration of 100 mM and diluted in culture medium to obtain the desired concentrations (2, 5, 10, and 50 μM). The final concentration of DMSO was kept below 0.02% to maximally eliminate the effect of DMSO. Control cells were incubated in the presence of 0.02% DMSO to compare the effect of 4,4′-diapotorulene. rBMSCs^49^ were seeded in a 48-well plate at a density of 3 × 10^3^ cells/well in Dulbecco’s modified Eagle’s medium (DMEM) supplemented with 10% fetal bovine serum (FBS), 100 units/mL penicillin, and 100 μg/mL streptomycin. After incubation for 24 h, the culture media were replaced with fresh DMEM supplemented with 10 ng/mL basic fibroblast growth factor and 20% FBS and further incubated overnight. Then, the media were replaced with induction media containing 4,4′-diapotorulene. 4,4′-Diapotorulene solution was prepared at concentrations of 2, 5, and 10 μM in culture medium. For controls, three wells were treated with culture media. At 1, 4, and 7 days, the viability of rBMSCs in the induction media was determined by using MTT [3-(4,5-dimethylthiazol-2-yl)-2,5-diphenyltetrazolium bromide], which was converted to formazan that accumulated in the cytoplasm of viable rBMSCs. The viability of rBMSCs was tested in three plates individually and the optical density of each well was determined at 590 nm using a plate reader (E-max, Molecular Device, USA). All experiments were performed at least three times and the results are presented as mean ± SD. To detect neuronal differentiation, digital images of the rBMSCs in the induction media containing 4,4′-diapotorulene were captured using an inverted phase-contrast microscope at 1, 4, and 7 days of incubation.

## Additional Information

**How to cite this article**: Kim, S. H. *et al*. Generation of structurally novel short carotenoids and study of their biological activity. *Sci. Rep.* **6**, 21987; doi: 10.1038/srep21987 (2016).

## Supplementary Material

Supplementary Information

## Figures and Tables

**Table 1:** Half-maximal effective concentration (IC_50_) values of C_30_ carotenoids towards DPPH.

	No. of conjugated double bonds	Functional group	DPPH IC_50_ (μM)
Diapolycopene	11	–	8.7
Diaponeurosporene	9	–	11.6
Diaponeurosporen-al	10	1 aldehyde	10.2
Diapolycopen-dial	13	2 aldehydes	7.5
Diaponeurosporenoic acid	10	1 carboxyl	9.7
Diapotorulene	8+1*	–	70.3
Diapo-β-carotene	5+2*	–	77.8
DL-α-tocopherol	–	–	33.2

^*^Additional double bond within the β-end group.
