# Supplementary material for: Generation of structurally novel short carotenoids and study of their biological activity
Source: Sci Rep. 2016 Feb 23;6:21987. doi: 10.1038/srep21987 (PMC4763220; doi:10.1038/srep21987)
Supplement: Supplementary Information [file srep21987-s1.pdf]

Supplementary Information

Generation of structurally novel short carotenoids and study of their biological activity

Se H. Kim[1], Moon S. Kim[2], Bun Y. Lee[2] & Pyung C. Lee[2,*]

[1]The Novo Nordisk Foundation Center for Biosustainability, Technical University of Denmark, Kogle Alle 6, 2970 Hørsholm, Denmark

[2]Department of Molecular Science and Technology and Department of Applied Chemistry and Biological Engineering, Ajou University, Woncheon-dong, Yeongtong-gu, Suwon 443-749, South Korea

[*]Address correspondence to Pyung Cheon Lee

E-mail: pclee@ajou.ac.kr, Tel: 82-31-219-2461, Fax: 82-219-1610

Table S1. Bacterial strains and plasmids used in this study

Strains and plasmids	Relevant properties	Source or reference
Strains		
Nostoc sp. PCC 7120	C_{40} carotenogenic pathway	UTEX 2576
Escherichia coli str. SURE	*endA*1 *glnV*44 *thi*-1 *gyrA*96 *relA*1 *lac recB recJ sbcC umuC*::Tn5 *uvrC e*14- Δ(*mcrCB-hsdSMR-mrr*)171 F'[*proAB*$^+$ *lacI*^{q} *lacZ*ΔM15 Tn10]	Stratagene
Escherichia coli str. XL1-Blue	*endA*1 *gyrA*96(*nal*^{R}) *thi*-1 *recA*1 *relA*1 *lac glnV*44 F'[::Tn10 *proAB*$^+$ *lacI*^{q} Δ(*lacZ*)M15] *hsdR*17(r_K^- m_K^+)	Stratagene

Pantoea agglomerans KCTC 2479	C_{40} zeaxanthin diglucoside biosynthesis pathway	KCTC
Rhodobacter capsulatus KCTC 2583	C_{40} spheroidenone biosynthesis pathway	KCTC
Corynebacterium glutamicum KCTC 1445	C_{50} decaprenoxanthin diglucoside biosynthesis pathway	KCTC
Salinibacter ruber DSMZ 13855	C_{40} carotenoid biosynthesis pathway	DSMZ
Plasmids		
pUCM	Cloning vector modified from pUC19. Constitutive *lac* promoter, Ap	1
pBBR1MCS-2	Cloning vector. SC101 origin. Inducible *lac* promoter, Km^R	2
pACYC184	Expression vector that is compatible with pMB1 or ColE1 related plasmids	NEB
pUCM-Y_{BL}	Constitutively expressed *crtYcYd* from *B. linens*	1
pUCM-Y_{tBL}	Constitutively expressed *crtYcYd* mutant genes	This study
pUCM-Y_{PA}	Constitutively expressed *crtY* from *P. agglomerans*	3
pUCM-Y_{PN}	Constitutively expressed *crtY* from *P. ananatis*	1
pUCM-Y_{SR}	Constitutively expressed *crtY* from *S. ruber*	This study
pUCM-Z_{PA}	Constitutively expressed *crtZ* from *P. agglomerans*	3
pUCM-X_{PA}	Constitutively expressed *crtX* from *P. agglomerans*	This study
pUCM-M_{SA}	Constitutively expressed *crtM* from *S. aureus*	4
pUCM-N_{SA}	Constitutively expressed *crtN* from *S. aureus*	4
pUCM-N_{rSA}	Constitutively expressed $crtN_r$ mutant	This study
pUCM-N_{ySA}	Constitutively expressed $crtN_y$ mutant	This study
pUCM-N_{zSA}	Constitutively expressed $crtN_z$ mutant	This study
pUCM-A_{RC}	Constitutively expressed *crtA* from *Rhodobacter capsulatus*	1
pUCM-D_{RC}	Constitutively expressed *crtD* from *Rhodobacter capsulatus*	This study
pUCM-Eb_{CG}	Constitutively expressed *crtEb* from *Corynebacterium glutamicum*	This study
pUCM-Y_{CG}	Constitutively expressed *crtYeYf* from *Corynebacterium glutamicum*	This study
pUCM-O_{SY}	Constitutively expressed *crtO* from *Synechocystis* sp. PCC6803	5
pUCM-W_{NO}	Constitutively expressed *crtW* from *Nostoc* sp. PCC7120	6
pBBR-aldH_{SA}	Inducibly expressed *aldH* from *S. aureus*	4

pACM-M_{SA}-N_{SA}	Constitutively expressed *crtM*, and *crtN* from *S. aureus* to produce 4,4′-diapolycopene	4
pACM-M_{SA}-N_{ySA}	Constitutively expressed *crtM*, and *crtN*$_y$ from *S. aureus* to produce 4,4′-diaponeurosporene	This study
pACM-M_{SA}-N_{zSA}	Constitutively expressed *crtM*, and *crtN*$_z$ genes from *S. aureus* to produce 4,4′-diapo-ζ-carotene	This study
pACM-M_{SA}-N_{SA}-P_{SA}	Constitutively expressed *crtM*, *crtN* and *crtP* genes from *S. aureus* to produce 4,4′-diaponeurosporen-4′-al	This study
pACM-M_{SA}-N_{ySA}-Y_{tBL}	Constitutively expressed *crtM*, *crtN*$_y$ and *crtY*$_t$ genes from *S. aureus* and *B. linens* to produce 4,4′-diapotorulene	This study
pACM-M_{SA}-N_{zSA}-Y_{PA}	Constitutively expressed *crtM*, *crtN*$_z$, and *crtY* genes from *S. aureus* and *P. agglomerans* to produce 4,4′-diapo-β-carotene	This study
pACM-M_{SA}-N_{ySA}-Y_{tBL}-Z_{PA}	Constitutively expressed *crtM*, *crtN*$_y$ *crtY*$_t$, and *crtZ* genes from *S. aureus*, *B. linens* and *P. agglomerans* to produce 7-hydroxy-4,4′-diapotorulene	This study
pACM-M_{SA}-N_{zSA}-Y_{PA}-Z_{PA}	Constitutively expressed *crtM*, *crtN*$_z$, *crtY*, and *crtZ* genes from *S. aureus* and *P. agglomerans* to produce 4,4′-diapo-β-cryptoxanthin	This study

Table S2. Primers used in this study

Gene	Sequence	Enzyme site
crtD$_{RC}$	F:5’ GCTCTAGAAGGAGGATTACAAAATGCGGAGTGAAACGGAC 3’	*Xba*I
	R:5’ CTGGCGATATCCTACTTCGCGGCGGAAATC 3’	*Eco*RV
crtEb$_{CG}$	F:5’ GCTCTAGAAGGAGGATTACAAAATGATGGAAAAAATAAGACTA 3’	*Xba*I
	R:5’ GGAATTCTTATATCTGATGAATTGCTAT 3’	*Eco*RI
crtY$_{CG}$	F:5’ GCTCTAGAAGGAGGATTACAAAATGATCCCTATCATCGATAT 3’	*Xba*I
	R:5’ GGAATTCCTACGGCTTTTCTGGCTC 3’	*Eco*RI
crtY$_{SR}$	F:5’ GCTCTAGAAGGAGGATTACAAAATGAGCTATCTATCTTTCCATC 3’	*Xba*I
	R:5’ GGAATTCTTAGGTCGCGGACAGG 3’	*Eco*RI
crtX$_{PA}$	F:5’ GCTCTAGAAGGAGGATTACAAAATGAGCCACTTTGCGGTC 3’	*Eco*RI
	R:5’ CCGGAATTCTCATACCGCGGCATAGTG 3’	*Not*I
Sub_HindIII_F	F:5’ CCCAAGCTTCCGACTGGAAAGCG 3’	*Hin*dIII
Sub_HindIII_R	R:5’ CCCAAGCTTCGGTGTGAAATACCG 3’	*Hin*dIII
Sub_BamHI_F	F:5’ CGGGATCCCCGACTGGAAAGCG 3’	*Bam*HI
Sub_BamHI_R	R:5’ CGGGATCCCGGTGTGAAATACCG 3’	*Bam*HI
Sub_SalI_F	F:5’ ACGCGTCGACCCGACTGGAAAGCG 3’	*Sal*I
Sub_SalI_R	R:5’ ACGCGTCGACCGGTGTGAAATACCG 3’	SalI
Sub_PpuMI_F	F:5’ ACGAGGACCCCCGACTGGAAAGCG 3’	*Ppu*MI
Sub_PpuMI_R	R:5’ ACGAGGACCCCGGTGTGAAATACCG 3’	*Ppu*MI
crtN A134V	F:5’ TAAAAAATATGAAATTGTACGTCGCTATTTCTTAG 3’	
	R:5’ CTAAGAAATAGCGACGTACAATTTCATATTTTTTA 3’	
crtN E246G	F:5’ ACTTAATGCTGAAATTGGGCAAATTATTATTGATC 3’	
	R:5’ GATCAATAATAATTTGCCCAATTTCAGCATTAAGT 3’	
crtN M58T	F:5’ CCCCACAATTGTCATGACGCCAGATGTTTATAAAG 3’	
	R:5’ CTTTATAAACATCTGGCGTCATGACAATTGTGGGG 3’	
crtN F443L	F:5’ CGAAATTTGGTTCGGCACTCGGTTTAATGCCAACT 3’	
	R:5’ AGTTGGCATTAAACCGAGTGCCGAACCAAATTTCG 3’	
crtN E209G	F:5’ AATTATTCCTATGATTGGAATGATGTTTGGTGTGC 3’	
	R:5’ GCACACCAAACATCATTCCAATCATAGGAATAATT 3’	
crtN N232S	F:5’ AGGGCTAGCGCAATTAAGTAAAGATTTAGGCGTTA 3’	
	R:5’ TAACGCCTAAATCTTTACTTAATTGCGCTAGCCCT 3’	
crtN F212S	F:5’ TATGATTGAAATGATGTCTGGTGTGCATTTTATTA 3’	
	R:5’ TAATAAAATGCACACCAGACATCATTTCAATCATA 3’	
crtN D350G	F:5’ AGGGCGATTATCGCATGGTCCTTCTATTTATGTGT 3’	
	R:5’ ACACATAAATAGAAGGACCATGCGATAATCGCCCT 3’	

Table S3. Nucleotide and amino acid changes in selected mutant clones

	Nucleotide	Codon usage	Amino acid
CrtNwt			
CrtNr04	C401T (GCA → GTA)	0.22 → 0.17	A134V
	A737G (GAG → GGG)	0.3 → 0.13	E246G
CrtNr07	A75G (GAA → GAG)	0.7 → 0.3	-
	A626G (GAA → GGA)	0.7 → 0.09	E209G
	A695G (AAT → AGT)	0.39 → 0.13	N232S
CrtNy02	T635C (TTT → TCT)	0.51 → 0.19	F212S
	T840C (AGT → AGC)	0.13 → 0.27	-
	A1049G (GAT → GGT)	0.59 → 0.38	D350G
CrtNy08	A185G (TAT → TGT)	0.53 → 0.43	Y62C
	T367C (TTT → CTT)	0.51 → 0.1	F123L
	T578G (ATT→ AGT)	0.47 → 0.13	I193S
	A627G (GAA → GAG)	0.7 → 0.3	-
	A1046G (CAT → CGT)	0.52 → 0.42	H348R
CrtNy11	T173C (ATG →ACG)	1.0 → 0.23	M58T
	T1327C (TTC → CTC)	0.49 → 0.1	F443L
	T1458C (AGT → AGC)	0.13 → 0.27	-

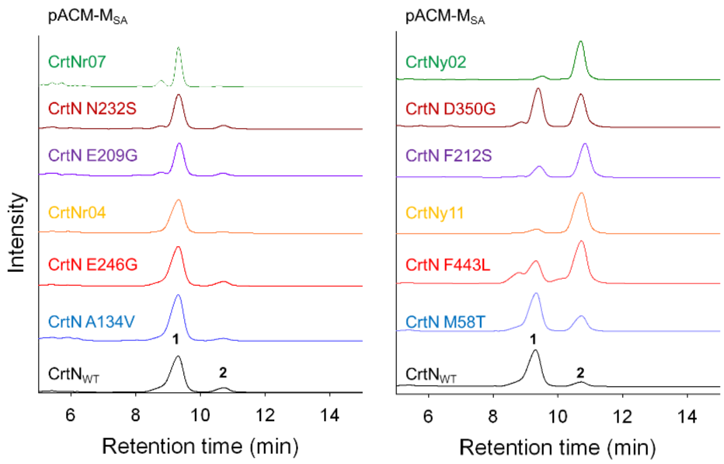

Figure S1. Carotenoid profiles of CrtN mutants. *E. coli* expressing background pACM-M_{SA} were transformed with each mutant clone, including mutants generated by site-directed mutagenesis and those cultivated for 48 h in TB medium. Carotenoids were identified as follows: 1. 4,4-diapolycopene, and 2. 4,4-diaponeurosporene.

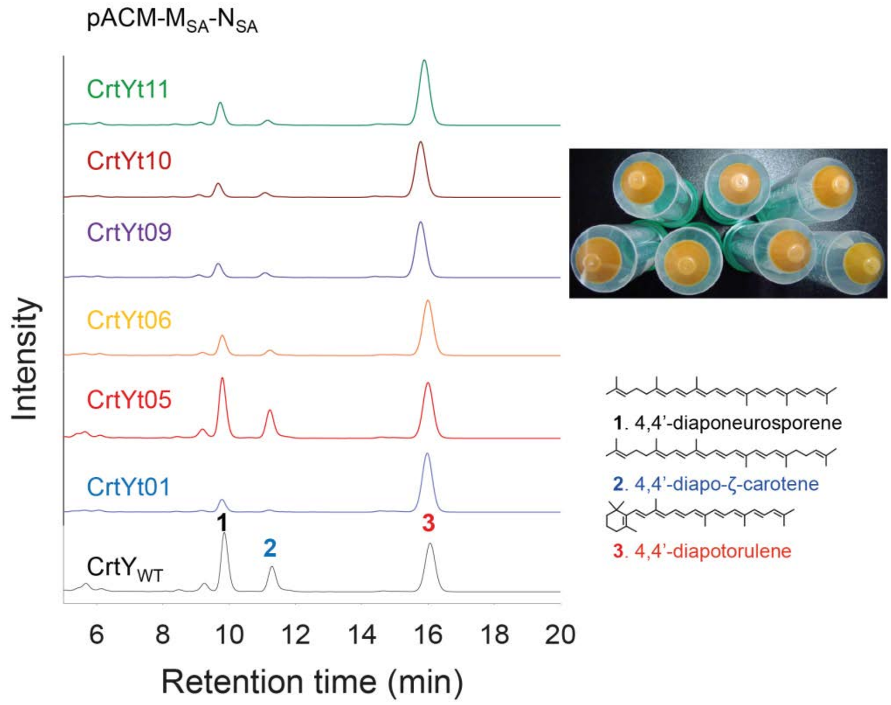

Figure S2. Carotenoid profiles of screened CrtY$_{BL}$ mutants. *E. coli* expressing background pACM-M$_{SA}$-N$_{SA}$ were transformed with each mutant clones and cultivated for 48 h in TB medium. Representative cell pellets and identified carotenoid structures are shown in the right panel.

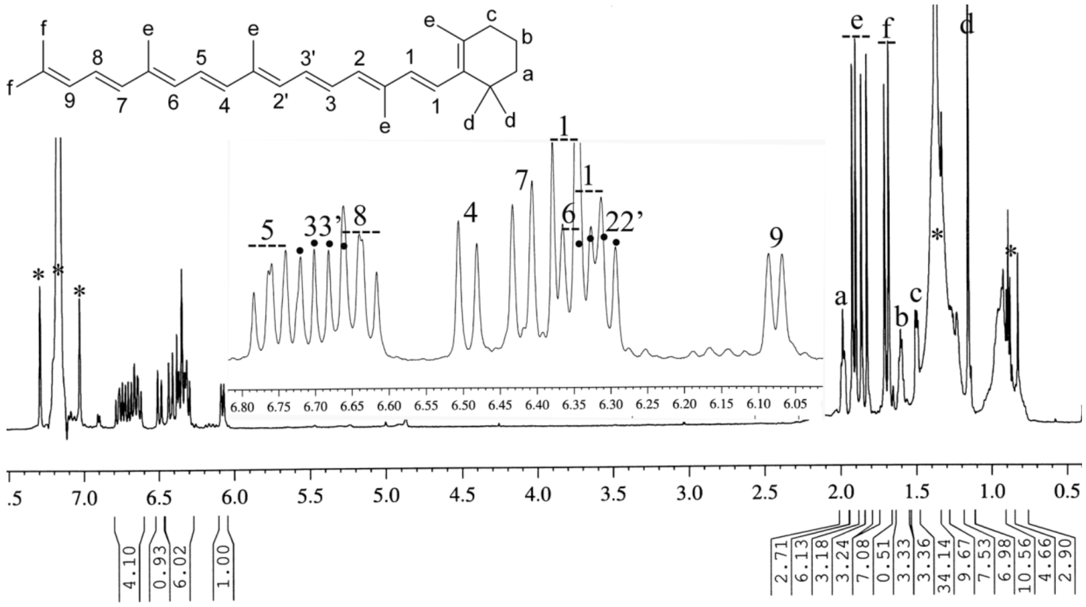

Figure S3. ^{1}H NMR spectrum of 4,4′-diapotorulene and its assignment. (The signals marked with "*" are from C_6D_6 and the eluent residue) ^{1}H NMR (600 MHz, C_6D_6): δ 6.76 (dd, *J* = 16, 11 Hz, 1H), 6.72, 6.70, 6.68, and 6.66 (AA'BB', 2H), 6.64 (dd, *J* = 16, 11 Hz, 1H), 6.49 (d, *J* = 16 Hz, 1H), 6.42 (d, *J* = 16 Hz, 1H), 6.362 (d, *J* = 19 Hz, 1H), 6.355 (d, *J* = 11 Hz, 1H), 6.329 (d, *J* = 19 Hz, 1H), 6.35, 6.33, 6.31, and 6.29 (AA'BB', 2H), 6.07 (d, *J* = 11 Hz, 1H), 1.98 (m, 2H), 1.92 (s, 3H), 1.90 (s, 3H), 1.86 (s, 3H), 1.83 (s, 3H), 1.71 (s, 3H), 1.68 (s, 3H), 1.60 (m, 2H), 1.50 (m, 2H), 1.16 (s, 6H) ppm.

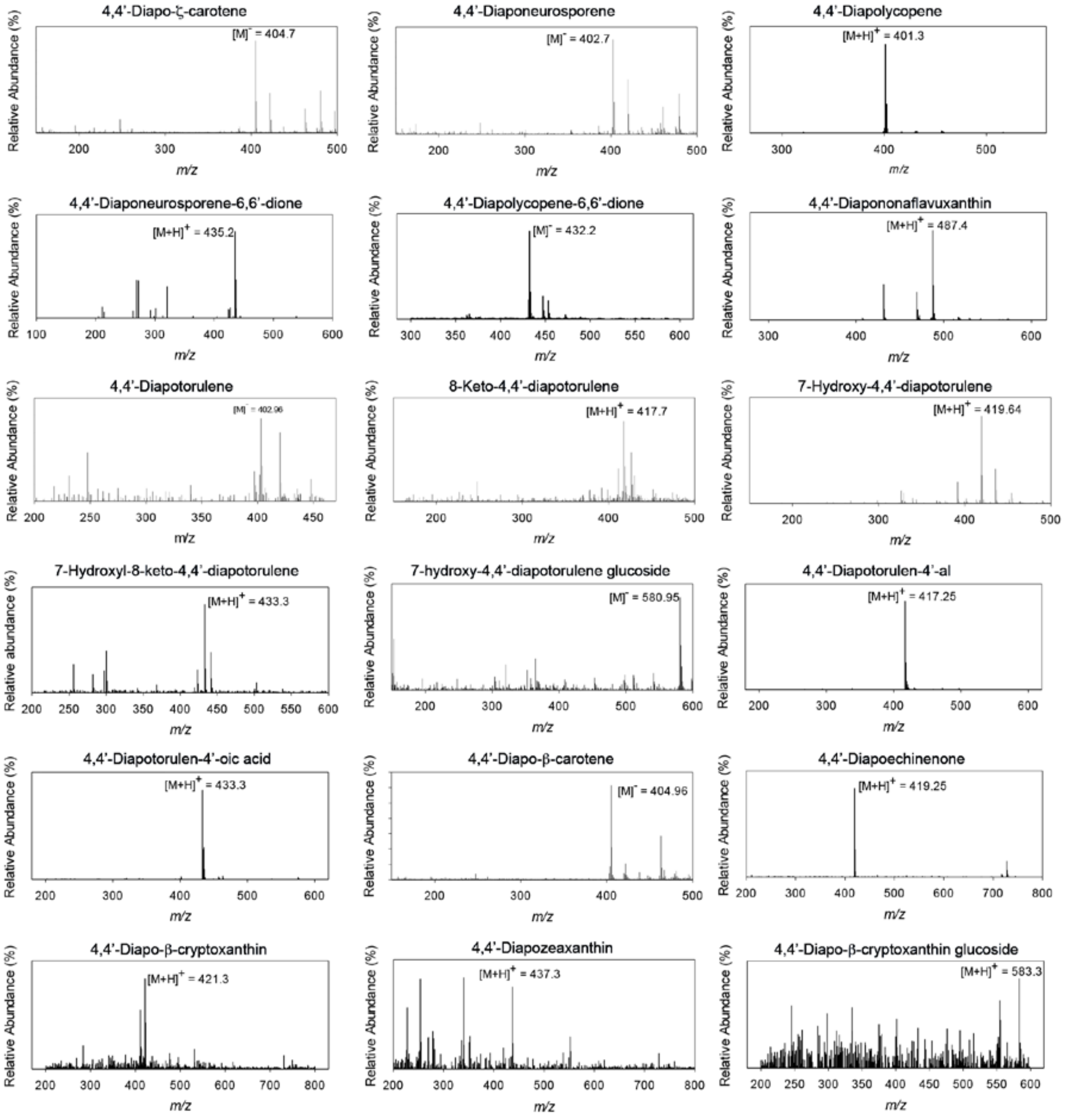

Figure S4. Mass spectra of carotenoids produced by engineered *E. coli* cells in this study. Mass spectra were recorded using both negative and positive APCI ion source as indicated in Materials and Methods.

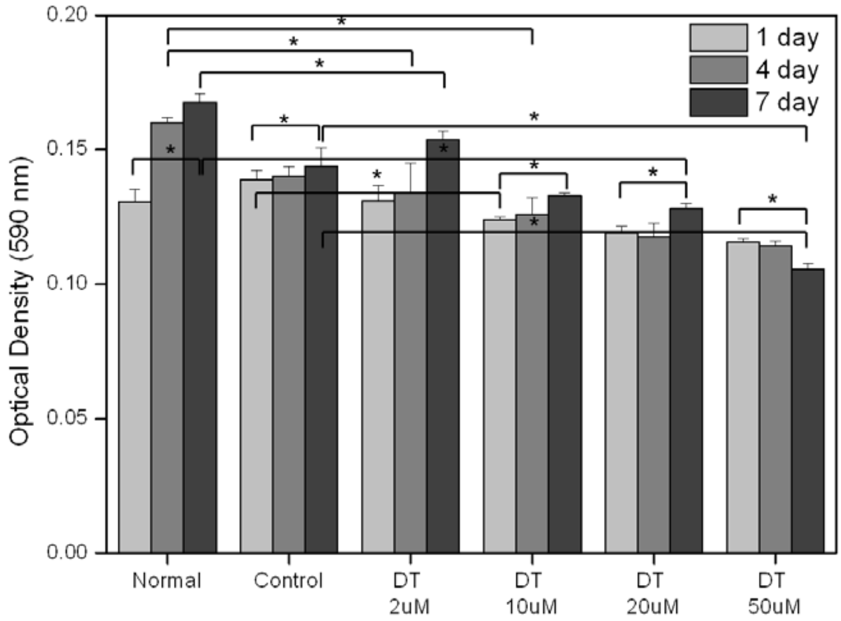

Figure S5. rBMSC viability after treatment with varying concentrations of 4,4′-diapotorulene for 7 days. Cells were treated with varying concentrations of 4,4′-diapotorulene, and the viability of the treated cells was measured with an MTT assay. Values are expressed as means ± SD and each experiment was performed in triplicate, (**p* < 0.001).

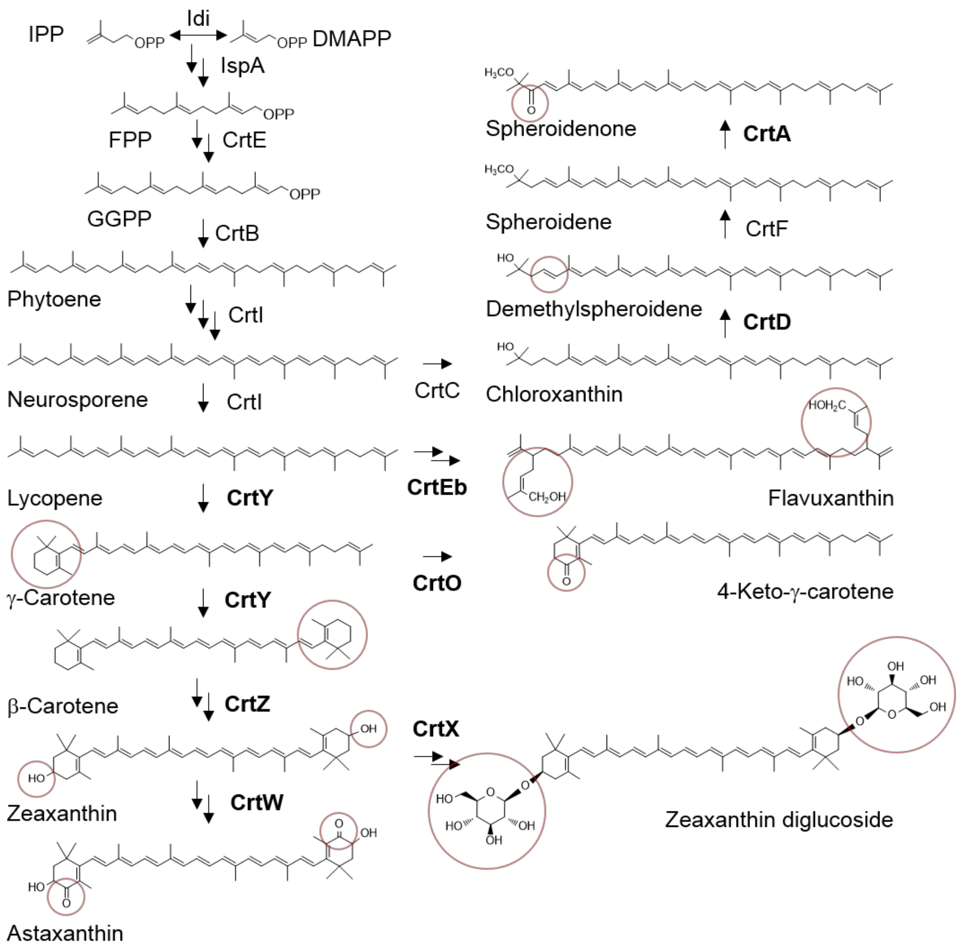

Figure S6. Selected C40 and C50 carotenoid biosynthesis pathway enzymes in nature. Enzymes represented in boldface were used for creating and extending structurally novel C30 and C35 carotenoids including acyclic, monocyclic, and bicyclic structures. Red circles indicate specific functional groups catalyzed by corresponding C40 or C50 carotenoid-modifying enzymes. Idi (IPP isomerase), IspA (FPP synthase), CrtE (GGPP synthase), CrtB (phytoene synthase), CrtI (phytoene desaturase), CrtY (lycopene cyclase), CrtZ (β-carotene hydrolase), CrtX (zeaxanthin glucosyltransferase), CrtW (β-carotene ketolase), CrtO (carotene ketolase), CrtEb (lycopene elongase), CrtD (1-hydroxycarotenoid 3,4-desaturase), and CrtA (spheroidene monooxygenase).

Supplementary References

1. Kim, S.H., Park, Y.H., Schmidt-Dannert, C. & Lee, P.C. Redesign, Reconstruction, and Directed Extension of the *Brevibacterium linens* C_{40} Carotenoid Pathway in *Escherichia coli*. *Appl. Environ. Microbiol.* **76**, 5199-5206 (2010).

2. Kovach, M.E. *et al.* Four new derivatives of the broad-host-range cloning vector pBBR1MCS, carrying different antibiotic-resistance cassettes. *Gene* **166**, 175-176 (1995).

3. Song, G.H., Kim, S.H., Choi, B.H., Han, S.J. & Lee, P.C. Heterologous carotenoid-biosynthetic enzymes: Functional complementation and effects on carotenoid profiles in *Escherichia coli*. *Appl. Environ. Microbiol.* **79**, 610-618 (2013).

4. Kim, S.H. & Lee, P.C. Functional expression and extension of staphylococcal staphyloxanthin biosynthetic pathway in *Escherichia coli*. *J. Biol. Chem.* **287**, 21575-21583 (2012).

5. Lee, P.C., Momen A.Z.R., Mijtz B.N. & Schmidt-Dannert, C. Biosynthesis of structurally novel carotenoids in *Escherichia coli*. *Chem. Biol.* **10**, 453-462 (2003).

6. Kim, S.H., Kim, J.H., Lee, B.Y. & Lee, P.C. The astaxanthin dideoxyglycoside biosynthesis pathway in *Sphingomonas* sp. PB304. *Appl. Microbiol. Biotechnol.* **98**, 9993-10003 (2014).
